# Editorial: Cardiovascular risk and lipoprotein(a): beyond LDL cholesterol

**DOI:** 10.3389/fcvm.2025.1607395

**Published:** 2025-05-29

**Authors:** Giuseppe Mandraffino, Laura D'Erasmo

**Affiliations:** ^1^ Unit of Internal Medicine, Department of Clinical and Experimental Medicine, University Hospital G. Martino, University of Messina, Messina, Italy; ^2^ Department of Translational and Precision Medicine, Sapienza University of Rome, Rome, Italy

**Keywords:** lipoprotein(a), Lp(a), atherosclerosis, cardiovascular risk factors (CVRFs), cardiovascular biomarkers, advancing knowledge

Editorial on the Research Topic Cardiovascular risk and lipoprotein(a): beyond LDL cholesterol

The role of lipoprotein(a) [Lp(a)] in cardiovascular disease (CVD) is increasingly recognized, yet it remains largely overlooked in routine clinical practice. While its pathophysiologic association with atherosclerosis and cardiovascular risk is well-documented, its precise contribution to various disease settings continues to be elucidated. Nowadays, evidence is accumulating about the association between Lp(a) levels and coronary heart disease, aortic valve stenosis, peripheral artery disease, ischaemic stroke, and cardiovascular but also all-cause mortality. New evidence linking Lp(a) levels to ischemic heart failure are now reported, notably by Zhang et al., suggesting Lp(a) as a standalone risk factor for MACE in these patients, also over LDL-C plasma levels.

Although awareness of Lp(a) and its role in cardiovascular disease has significantly increased in Western countries to date, there remain many areas outside mainstream healthcare systems where the contribution of Lp(a) to patients’ health is either underestimated or entirely unconsidered. For instance, the study by Hillmeister et al., which focused on a representative cohort from a non-metropolitan region in Germany, uncovered a previously unknown high prevalence of elevated Lp(a) levels among cardiovascular patients, many of whom had never been tested for Lp(a). This was largely due to a lack of awareness of Lp(a) as a risk factor for CVD.

The growing body of evidence supporting the role of Lp(a) in cardiovascular disease is helping to raise global awareness. This is highlighted by the INTERHEART study from the Middle East-Gulf region, which enrolled 595,658 subjects. Manla et al. reported that the rate of Lp(a) testing doubled from 2017 to 2022, although it still remained low, with only one in fifty enrolled patients being tested.

Given this evidence, one might assume that a large number of patients under the care of cardiologists or endocrinologists would have undergone at least one Lp(a) test. However, the proportion of specialists who have never tested their patients for Lp(a) is surprisingly high. A recent report from Singapore by Loh et al. confirmed that the lack of effective treatment is the most common barrier to testing.

Despite the accumulating evidence supporting the role of Lp(a) in cardiovascular disease and coronary artery vasculopathy, its integration into clinical practice remains inconsistent. Although Lp(a) testing is now recommended at least once in a lifetime, it is still rarely performed in many healthcare settings, even among high-risk individuals. While several therapeutic strategies are under evaluation, until targeted treatments are widely available, aggressive management of other cardiovascular risk factors remains the most effective approach to mitigating the risks associated with elevated Lp(a).

We believe that the future of lipidology must extend beyond LDL-C, focusing also on the atherogenicity and valvulopathy associated with Lp(a). Universal Lp(a) screening, enhanced clinician awareness, and the rapid development of targeted therapies aimed at Lp(a) should be key priorities in reducing residual cardiovascular risk and improving outcomes for patients with elevated Lp(a).

